# Preliminary Observations on Sensitivity and Specificity of Magnetization Transfer Asymmetry for Imaging Myelin of Rat Brain at High Field

**DOI:** 10.1155/2015/565391

**Published:** 2015-08-27

**Authors:** Jae-Woong Kim, Jiye Choi, Janggeun Cho, Chulhyun Lee, Daejong Jeon, Sung-Hong Park

**Affiliations:** ^1^Department of Bio and Brain Engineering, Korea Advanced Institute of Science and Technology, Daejeon 305-701, Republic of Korea; ^2^Division of Magnetic Resonance Research, Korea Basic Science Institute, Ochang-eup, Cheongwon-gun, Chungcheongbuk-do 363-883, Republic of Korea; ^3^Department of Chemistry, Chung-Ang University, Seoul 156-756, Republic of Korea

## Abstract

Magnetization transfer ratio (MTR) has been often used for imaging myelination. Despite its high sensitivity, the specificity of MTR to myelination is not high because tissues with no myelin such as muscle can also show high MTR. In this study, we propose a new magnetization transfer (MT) indicator, MT asymmetry (MTA), as a new method of myelin imaging. The experiments were performed on rat brain at 9.4 T. MTA revealed high signals in white matter and significantly low signals in gray matter and muscle, indicating that MTA has higher specificity than MTR. Demyelination and remyelination studies demonstrated that the sensitivity of MTA to myelination was as high as that of MTR. These experimental results indicate that MTA can be a good biomarker for imaging myelination. In addition, MTA images can be efficiently acquired with an interslice MTA method, which may accelerate clinical application of myelin imaging.

## 1. Introduction

Myelin is an essential microstructure component for normal brain function. It is a type of glial cells that wraps around axons, forming dielectric myelin sheath structure. Myelin induces saltatory conduction enabling nerve impulse to propagate more than 200 times faster. Moreover, myelin accounts for 50% of the dry weight of central nervous system [1]. For these reasons, imaging and observing myelination state are important to understand neurological state and diagnose neural diseases. The gold standard to image myelination is direct observation using staining [2]. Various histochemistry methods have been developed and verified by comparing normal and impaired brains. However, the methods are not applicable to clinical studies due to invasiveness. Medical imaging devices, therefore, have drawn neurological and neuropathological interests for noninvasive clinical diagnosis of myelin-related diseases such as multiple sclerosis (MS) [3–7] and schizophrenia [8–11].

Magnetic resonance imaging (MRI) provides apparent soft tissue contrast and various approaches have been developed for imaging myelin distribution using MRI. Myelin water imaging is an approach that utilizes short *T*_2_ decay of water in myelin sheath [12, 13]. The method assumes the measured decay curve to be weighted sum of multiexponential functions. A nonnegative least square algorithm plays a crucial role in converting the decay curves to *T*_2_ distribution and thus clearly separating myelin water signal from the other tissue water signals [5, 12–17]. However, long scan time per slice and long data processing time hinder its clinical applications. Other approaches for separation of myelin from other components based on faster decay of myelin are *T*_2_^*∗*^-based methods [7, 18] and a double inversion recovery method [19].

Another fast and efficient MRI approach for myelin imaging is magnetization transfer (MT). MT is a phenomenon of magnetization exchange between free water and proton bound to macromolecules. The exchange enables an RF pulse of off-resonance saturation to decrease signals in free water typically used in MR imaging, thereby indirectly providing information of proton bound to macromolecules. MT ratio (MTR) is typically measured for the MT techniques and has been used for appraising abnormality of brain structures, especially those related to myelination. MTR imaging offers noticeable sensitivity on white matter (WM), showing decreased signal on the myelinated regions in multiple sclerosis (MS) patients [4, 6, 20–22] and demyelinated animals [23–26]. The MTR method has advantages of shorter scan time and shorter postprocessing time than myelin water imaging. However, the MTR signal is not exclusively high in myelinated tissue, indicating low specificity. In other words, MTR can be generated by various pathophysiological components. The water concentration in multiple sclerosis lesions is induced by inflammatory activity and affects the MTR signal [24, 27]. Also, densely packed structure of biological tissue (i.e., skeletal muscle) has plenty of macromolecular bound protons resulting in high MTR signal [28].

In previous studies, the asymmetric MT effects around the water-resonance frequency have been reported [29–31]. Recently, a study proposed a possibility of imaging myelination by using asymmetric chemical exchange saturation transfer effect in the domain of Nuclear Overhauser Effects [32]. Another previous study directly measured extracted myelin in ^1^H NMR system and reported that myelin exhibits main peak at 3.5 ppm upfield from water-resonance frequency due to the chemical shift [33]. From these results, it is anticipated that taking advantage of the chemical shift as well as the MT strength (rather than the MT strength alone) would be beneficial to image myelination. This notion indicates that MT asymmetry (MTA) may be a better choice than the conventional MTR approach for improving the specificity of the myelin signals, by suppressing the MT signals from the nonmyelin tissues.

The goal of this study is to assess the sensitivity and specificity of the MTA approach for imaging myelination. We hypothesized that MTA imaging would provide better specificity to myelin than conventional MTR. For the MTA and MTR imaging, we used a new method, alternate ascending/descending directional navigation (ALADDIN), which enables us to acquire interslice perfusion-weighted and MTA images simultaneously [34–37], and MTR imaging was also performed through ALADDIN by additionally acquiring MT free images [38]. We investigated the contrast between WM and other brain tissues on various flip angles and compared the results of MTA to those of MTR. We also examined MTA and MTR in normal rats and demyelinated rats through the histogram analysis, which is typically used in the MTR studies [3, 4, 39, 40], in order to investigate the effects of the myelination state on the two MT measures of MTA and MTR.

## 2. Materials and Methods

### 2.1. Animal Preparation

Total 6 male Sprague-Dawley rats with 8-9 weeks of age were scanned in this study approved by the Institutional Animal Care and Use Committee at the Korea Basic Science Institute. Before MRI scan, rats were initially anesthetized by 5% isoflurane mixed with a 3 : 7 mixture of pure oxygen and nitrous oxide in a plastic box. The animals were then fixed in a cradle to minimize motion and the isoflurane level was decreased at 1.5% for anesthesia with a breath mask. The body temperature was maintained at around 37°C. After being placed inside the MRI system, the animals were monitored for the breath rate to be kept at 50–70 per minute by adjusting the isoflurane level at around 1.5%. To examine demyelination effects, 4 rats (2 control and 2 demyelinated rats) with 8 weeks of age were used for the MRI scans. For the 2 rats of demyelination, 0.2% (w/w) cuprizone (bis-cyclohexanone oxaldihydrazone, Sigma-Aldrich Inc., St. Louis, MO, USA) mixed into a ground standard rodent chow from 4 weeks to 8 weeks of age [41]. In order to study remyelination effects, the cuprizone administration was stopped for the demyelinated rats from 8 weeks of age (the first MRI study) to 9 weeks of age (for one week) and then the second MRI study was performed for the remyelinated rats.

### 2.2. Data Acquisition

**Figure 1:**
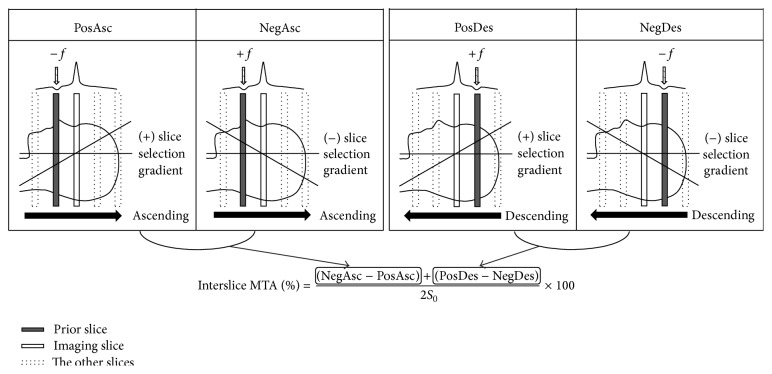
Alternate ascending/descending directional navigation (ALADDIN) data acquisition and reconstruction schemes used in this study. Four different types of data acquisition were sequentially performed by alternating the slice acquisition order (ascending and descending, “Asc” and “Des”) and the slice-selection gradient polarity (positive and negative, “Pos” and “Neg”). The on-resonance RF pulses for imaging the prior slices simultaneously act as off-resonance saturation pulses for the present imaging slice. *f* indicates the saturation offset frequency induced by the first prior slice. The four types of images (PosAsc, NegAsc, PosDes, and NegDes) are combined to generate an interslice MT asymmetry (MTA) image. *S*_0_ represents the average of the four types of images.

All experiments were conducted on a 9.4 T Varian animal MRI system (Palo Alto, CA, USA) with a volume RF coil (a diameter of 72 mm) for both transmission and reception of the signals. All the MTA and MTR acquisitions were performed using ALADDIN based on balanced steady state free precession (bSSFP) readout, as described previously at low fields [34–36, 38]. Briefly, 4 different types of data were acquired by alternating the slice acquisition order (i.e., ascending and descending) and the slice-select gradient polarity (denoted as PosAsc, NegAsc, PosDes, and NegDes in Figure 1). The MTA was acquired by combining the 4 datasets to separate interslice MTA out of the interslice blood flow effects, as described in Figure 1 and also in the references [34, 36]. The readout gradient polarity was also alternated for averaging to suppress the effects of gradient imperfections, and thus eventually total 8 different types of data were acquired [34, 36].

#### 2.2.1. Flip Angle-Dependent Study

To investigate the specificity of magnetization transfer signals of WM relative to the other brain tissues, 2 healthy male rats with 8-9 weeks of age were scanned using ALADDIN at various flip angles of 30°, 45°, and 60°. The imaging parameters were TR = 3.8 ms, TE = 1.9 ms, matrix size = 128 × 128, field of view = 30 × 30 mm^2^, number of slices = 21, phase cycle angle = 180°, slice thickness = 1 mm, phase over sampling = 0%–100%, and total scan time = 8.2–13.6 min. A large interslice gap (100% of the slice thickness) was used to avoid the crosstalk effects. The interslice offset frequency for the first prior slice was 6700 Hz corresponding to 16.7 ppm at 9.4 T. For MTR imaging, MT free acquisition was performed with interslice delay of 6 seconds for longitudinal magnetizations to recover back to the equilibrium state. The MT free scan was averaged once. MTR images were calculated using the formula: MTR (%) = (*S*_0_ − *S*_MT_)/*S*_0_ × 100, where *S*_0_ and *S*_MT_ stand for MT free and MT weighted images. *S*_MT_ was obtained by averaging the images from both positive and negative offset frequencies.

#### 2.2.2. Demyelination and Remyelination Effects on MT Signals

The two demyelinated rats and the two control rats were scanned at 8 weeks of age. The remyelinated rats were scanned one week after the first MRI scan (i.e., 9 weeks of age). The scan parameters were similar to the above flip angle-dependent study except TR = 3.8–4.4 ms, TE = 1.9–2.2 ms, bandwidth = 64–100 kHz, matrix size = 128 × 128, flip angle = 60°, phase oversampling = 0%, and scan time per dataset = 31.1–37.8 min. The ALADDIN acquisitions were averaged 24 times, resulting in the number of repeated acquisitions of volume to be 192. Because of the long scan time, the water-resonance frequency was recalibrated every 3 repeated volume acquisitions to minimize *B*_0_ drift effects. MT free scan was averaged 4 times with interslice delay of 5 s.

### 2.3. Data Processing and Analysis

**Figure 2:**
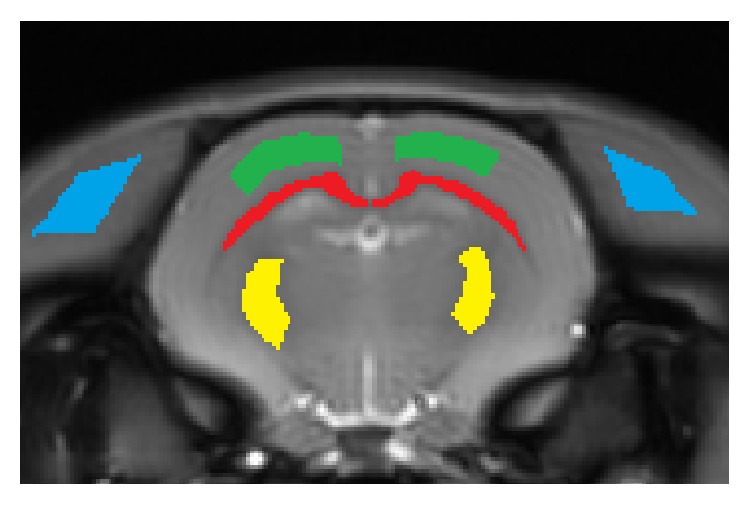
Regions of interest (ROIs) manually defined in each hemisphere from a baseline image. Red: corpus callosum (CC), yellow: internal capsule (IC), green: gray matter (GM), and blue: muscle. White matter (WM) ROI is combination of CC and IC.

For each rat, we selected a slice of interest that included both corpus callosum (CC) and internal capsule (IC). The regions of interest (ROIs) were manually defined in CC, IC, GM, and muscle areas from each hemisphere using MATLAB, avoiding regions of banding artifacts (*N* = 4 hemispheres for each group).  Figure 2 presents the exemplary ROIs and slice of interest in a rat brain. All statistical analyses were performed based on the average intensities within the ROIs by using one-tailed nonparametric tests at the significance level of *α* = 0.1 because of the small sample size.

In the flip angle-dependent study, average intensities were calculated within WM (CC + IC), GM, and muscle ROIs and then plotted as a function of flip angle. Subsequently, specificity was evaluated based on the definition that specificity is the proportion of negatives that are correctly measured. If a technique is prominently sensitive only to WM but not to tissues with no myelination (e.g., muscle), then it is considered having high specificity to myelination. The statistical significance was assessed between WM and GM as well as between WM and muscle on each flip angle by using the Wilcoxon signed rank test.

In the study of demyelination and remyelination effects on the MT signals, histograms were obtained from the ROIs and normalized by the number of pixels within the ROI. Subsequently, the histograms of MTA and MTR from demyelinated rats and control rats were compared in regard to histogram shape, mean value, and standard deviation within the ROIs. Because most of the MTA and MTR signals were less than 15% and 45%, respectively, MTA and MTR histograms were plotted within ranges of 0%–15% and 0%–45%, respectively, with the same number of bins. Sensitivity was assessed for the demyelination and remyelination study. Sensitivity is the proportion of positives that are correctly measured. If changes in the myelin contents through demyelination and remyelination can be detected, the technique is considered having high sensitivity to myelination. Therefore, the statistical significance was assessed between demyelinated and control rats and between remyelinated and control rats in both WM and GM by using the Wilcoxon rank-sum test.

## 3. Results

### 3.1. Flip Angle-Dependent Study

**Figure 3:**
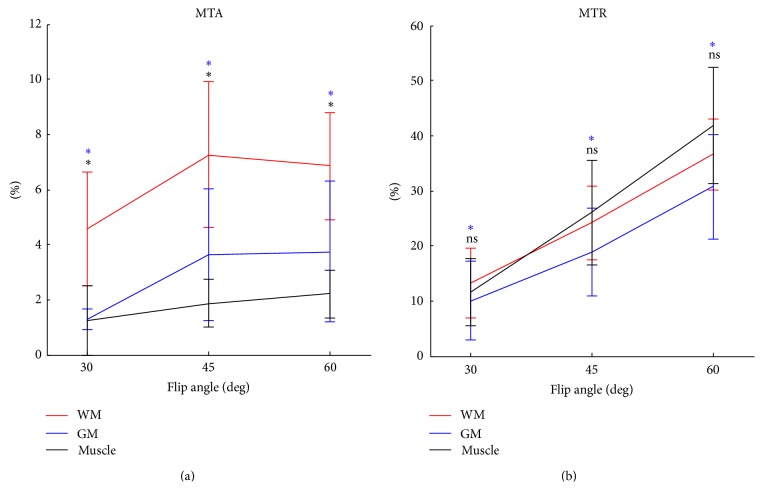
MT asymmetry (MTA) and MT ratio (MTR) plots as a function of flip angle. The error bars represent the range of one standard deviation. The blue and black asterisks (upper and lower rows, resp.) represent the significant differences between WM and GM and between WM and muscle, respectively. *∗*: *p* < 0.1, ns: *p* ≥ 0.1.

**Figure 4:**
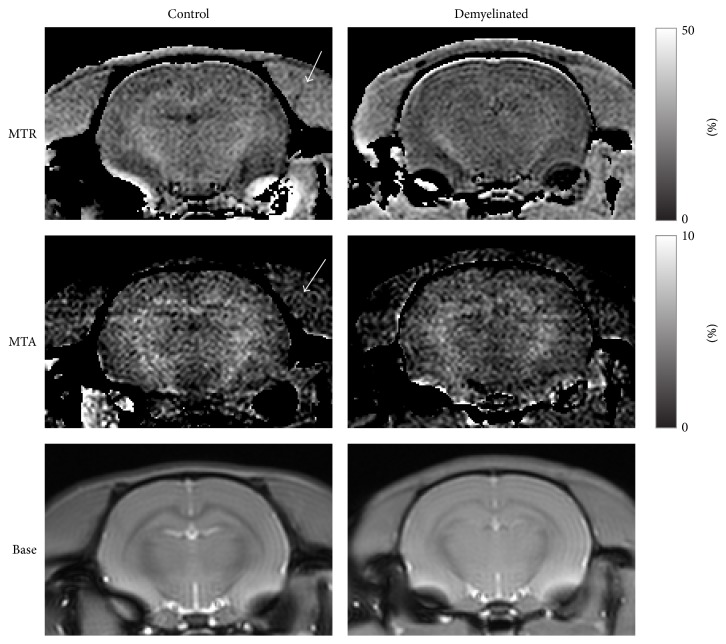
Representative MT asymmetry (MTA), MT ratio (MTR), and baseline (Base) images. Left and right columns correspond to images of control rats and demyelinated rats, respectively. The white arrow indicates the muscle region, where MTA and MTR signals are suppressed and enhanced, respectively.

Figure 3 shows the MTA and MTR signal intensities, which generally increased with flip angle in most cases. However, there were differences in the results of the ROI analysis between MTA and MTR. The MTA signals showed plateau or even decreased at 60°, whereas the MTR signals continuously increased with flip angle. MTA showed much higher WM signals than GM and muscle, while MTR showed WM signals as high as muscle signals. The statistical tests showed that MTA discriminated WM from the other tissues, but MTR showed difference only between WM and GM (Figure 3), indicating that MTA is more specific to myelinated tissue than MTR. The visual inspection of the MTA and MTR images showed similar results (Figure 4). In the MTR images (the first row in Figure 4), WM signals were strong; however, GM and muscle signals were also not negligible. The MTA images (the second row of Figure 4) also showed higher intensities in WM but noticeably suppressed signals in the other tissue regions.

### 3.2. Demyelination and Remyelination Effects on MT Signals

**Figure 5:**
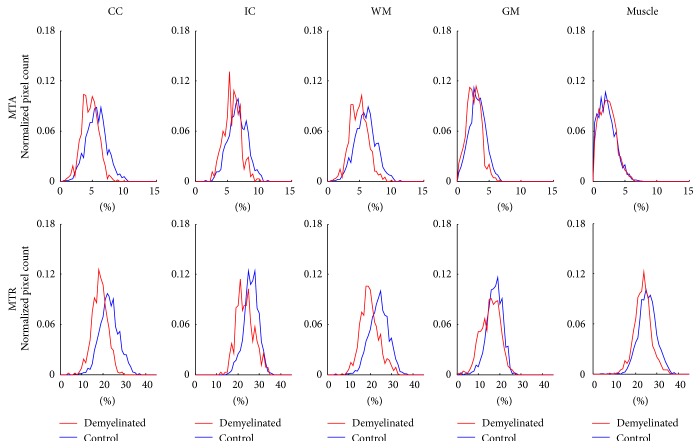
Normalized histogram results from demyelinated rats and control rats showing demyelination effects. CC: corpus callosum, IC: internal capsule, WM: white matter (CC + IC), and GM: gray matter.

**Figure 6:**
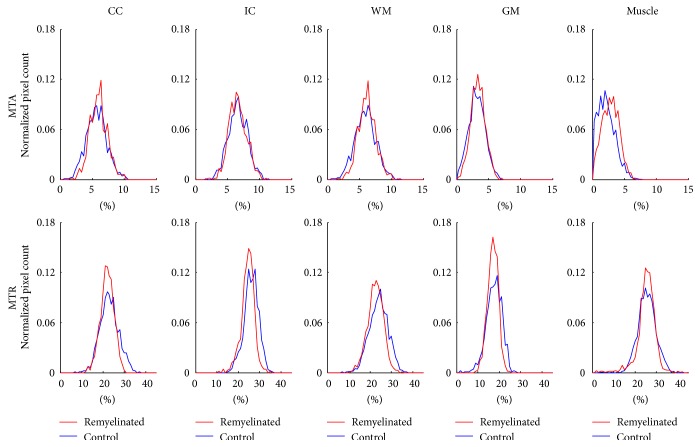
Normalized histogram results from remyelinated rats and control rats showing remyelination effects. CC: corpus callosum, IC: internal capsule, WM: white matter (CC + IC), and GM: gray matter.

Figures 5 and 6, respectively, show histograms from tissue regions of demyelinated and remyelinated rats in comparison with those of control rats. For both MTA and MTR, the histograms of demyelinated rats shifted left compared to those of the control rats, indicating signal decrease due to demyelination effects. In contrast to the results of demyelinated rat brains, the histograms from the remyelinated rats showed almost no difference from those of the control rats in both MTA and MTR (Figure 6), indicating signal recovery due to the remyelination effects.

**Figure 7:**
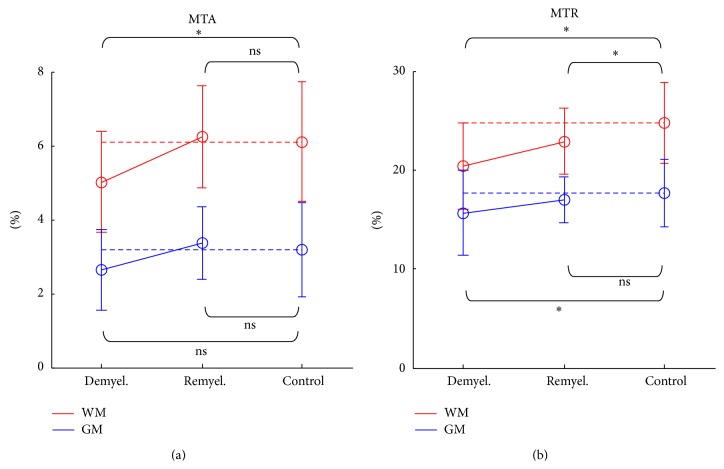
MT asymmetry (MTA) (a) and MT ratio (MTR) (b) values for demyelinated (Demyel.), remyelinated (Remyel.), and control rats. MTA and MTR are plotted in different intensity scales. WM: white matter, GM: gray matter. *∗*: *p* < 0.1, ns: *p* ≥ 0.1.

The quantitative MTA and MTR values for the demyelinated, remyelinated, and control rats (Figure 7) were roughly in agreement with the histogram analysis. Both MTA and MTR commonly show signal decrease from demyelination and signal increase from remyelination. However, the statistical results showed that MTA signal changes were significant only for WM whereas MTR signal changes were significant for both WM and GM except GM in the remyelinated rat brain (Figure 7).

## 4. Discussion

The ROI analysis of the flip angle-dependent study and the visual inspection of the representative images indicate that MTA provides better WM specificity than MTR (Figures 3 and 4). Also, the demyelination and remyelination studies indicate that MTA has sensitivity to myelination as high as MTR (Figures 5–7). The MTA signal is derived from asymmetric MT spectrum, indicating that the strength of MT saturation effect is not symmetric around the water-resonance frequency. According to the previous studies, the saturation is stronger at the negative offset frequency (−*f*) than the positive offset frequency (+*f*), because of the negatively shifted MT spectrum of macromolecular proton pool in the central nervous system [11, 29–31].

High MTA can be achieved when the two conditions are satisfied: (i) the shift in the center of bound proton pool MT spectrum compared to free proton pool and (ii) strong MT saturation which depends on the tissue type. As shown in the result part, MTA exhibited noticeably high signals in WM and low signals in the other brain tissues. We can postulate that the distinction of WM in MTA method is due to a certain component prevalent in WM, but rare in GM and muscle. In the brain, myelin is a leading candidate for the main signal source of MTA, because myelin satisfies the two conditions above. First, myelin is mostly made up of lipid which forms a main peak centered at about 3.5 ppm upfield from water-resonance frequency in ^1^H NMR system due to the chemical shift [33]. About 80% of myelin is lipid and ~20% is protein [42]. The lipid composition is much higher in WM (49–66%) than in GM (36–40%). Therefore, the exchange of magnetizations takes place mostly between water protons and lipid-bound aliphatic protons which compose the side chains of myelin. The interaction between free proton pool and aliphatic proton pool relates to Nuclear Overhauser Effects and provides broad contribution to the MT spectrum around −3.5 ppm [32, 43]. Second, from previous studies about MTR, myelin is known to be sensitive to MT-related imaging techniques indicating that myelin has MT effects as high as or even stronger than those in the other brain tissues [39, 44, 45]. As a result, it is plausible that myelin property of the high lipid composition induces not only shift in MT spectrum of bound proton pool but also vast contribution of MT saturation, resulting in high MTA signal.

Sensitivity of MTA to myelination was demonstrated to be similar to that of MTR through the demyelination and remyelination studies (Figures 5–7). MTA successfully detected the change of myelination state caused by the cuprizone administration (Figure 5) and one week interval of no cuprizone administration (Figure 6) in the same manner as MTR, which is in agreement with the other MTR studies [25, 26, 46]. In this study, the MTR signal loss from cuprizone-induced demyelination was lower than that in other studies [25, 46, 47] and the MTR recovery from remyelination was relatively faster compared to the results of other studies [25, 26, 46]. It could be due to the fact that the dose of cuprizone in this study (0.2%), which has been typically adopted for demyelination of mice rather than rats [25, 26, 46, 48–50], was relatively lower than the dose used in the other studies for demyelination of rats [51, 52]. The remyelination effects observed in this study after one week of no cuprizone administration were generally in agreement with the previous studies, which reported significant remyelination shortly after the termination of cuprizone diet [48, 49, 53, 54].

Small signal changes in GM were observed with demyelination and remyelination for MTR (Figure 7). Similar signal changes in GM were also observed for MTA in the histogram analysis, despite being statistically not significant (Figure 7). This could be ascribed to the recovery of small amount of myelin in GM [55]. The oral administration of cuprizone might induce a global demyelination regardless of tissue types. The cuprizone-induced demyelination in cerebral cortex is found in another animal study with decrease of immunodetection [52]. Decreased MTR signal in GM was also reported in previous studies about MS patients [40, 56–58]. In a previous study with demyelinated and remyelinated mice [26], the MTR signal changes in deep GM reached statistical significance but those in the cerebral cortex (similar to GM in this study) were not significant, an observation slightly different from this study. Therefore, further studies are necessary to assess the significance of signal changes in GM associated with demyelination and remyelination.

The high MTR signal from muscle may be attributed to the unique structure of the muscle tissue. Muscle has numerous micromuscle fibers aligned in parallel that are surrounded by electrolyte, providing a suitable environment for transfer of magnetization. However, unlike the case of myelin, the MT effect does not mainly occur between lipid proton pool and free proton pool, resulting in mostly symmetric MT spectrum and thus low MTA signals.

When bSSFP readout is combined to interslice imaging method based on ALADDIN, the scan time can be effectively reduced because of the absence of MT presaturation period, which was already demonstrated in the clinical scanner [34, 36]. This advantage of ALADDIN may potentially facilitate clinical application of MTA in a reasonable scan time.

## 5. Conclusion

In this study, MTA was compared to conventional myelin imaging technique, MTR, in terms of imaging myelination. MTA imaging revealed high signal in WM and significantly low signals in GM and muscle, indicating better specificity than MTR. The demyelinated rats revealed apparent decrease of MTA and MTR signals, whereas remyelinated rats showed enhanced MTA and MTR signals comparable to those of normal rats. The main signal source of MTA in WM is presumed to be the MT effects occurring between free proton pool and lipid-bound proton pool associated with myelination. The experimental results indicate that MTA can be a good biomarker for imaging myelination with better specificity than and similar sensitivity to MTR. In addition, MTA images can be efficiently acquired with ALADDIN, which may accelerate clinical application of myelination imaging.
